# Effect of Plasma and Blood Donations on Levels of Perfluoroalkyl and Polyfluoroalkyl Substances in Firefighters in Australia: A Randomized Clinical Trial

**DOI:** 10.1001/jamanetworkopen.2022.6257

**Published:** 2022-04-08

**Authors:** Robin Gasiorowski, Miriam K. Forbes, Gabriel Silver, Yordanka Krastev, Brenton Hamdorf, Barry Lewis, Michael Tisbury, Merrole Cole-Sinclair, Bruce P. Lanphear, Roger A. Klein, Nigel Holmes, Mark Patrick Taylor

**Affiliations:** 1 Faculty of Medicine, Health and Human Sciences, Macquarie University, New South Wales, Australia; 2 Department of Haematology, Concord Repatriation General Hospital, New South Wales, Australia; 3 Centre for Emotional Health and School of Psychological Sciences, Macquarie University, New South Wales, Australia; 4 Now with Research, Innovation & Enterprise, Office of the Deputy Vice Chancellor, Research, Macquarie University, New South Wales, Australia; 5 High Consequence Chemical Response Capability Project, Fire Rescue Victoria (FRV), Victoria, Australia; 6 FRV Advocacy, FRV, Victoria, Australia; 7 Laboratory Haematology Department, St Vincent’s Hospital, Melbourne, Victoria, Australia; 8 Faculty of Health Sciences, Simon Fraser University, Vancouver, British Columbia, Canada; 9 Retired, Cambridge, United Kingdom; 10 Christian Regenhard Center for Emergency Response Studies, City University of New York, New York; 11 Incident Response Unit, Environmental Services and Regulation, Queensland Department of Environment and Science, Queensland, Australia; 12 Environment Protection Authority Victoria, EPA Science, Centre for Applied Sciences, Macleod, Melbourne, Victoria, Australia; 13 Earth and Environmental Sciences, Faculty of Science & Engineering, Macquarie University, New South Wales, Australia

## Abstract

**Importance:**

Elevated levels of blood perfluoroalkyl and polyfluoroalkyl substances (PFASs) have been associated with a range of adverse health outcomes. Firefighters have been exposed to PFASs in firefighting foams and have previously been shown to have higher PFAS levels in blood samples than the general population. No interventions have been shown to reduce PFAS levels.

**Objective:**

To examine the effect of blood or plasma donations on PFAS levels in firefighters in Australia.

**Design, Setting, and Participants:**

This 52-week, open-label, randomized clinical trial enrolled participants from May 23 to August 23, 2019. Participants were 285 Fire Rescue Victoria staff or contractors with serum levels of perfluorooctane sulfonate (PFOS) of 5 ng/mL or more who were eligible to donate blood, had not donated blood in the 3 months prior to randomization, and were able to provide written informed consent. Analysis was performed on an intention-to-treat basis from May to July 2021.

**Interventions:**

Firefighters with baseline PFOS levels of 5 ng/mL or more were randomly assigned to donate plasma every 6 weeks for 12 months, donate blood every 12 weeks for 12 months, or be observed only.

**Main Outcomes and Measures:**

The primary end points were changes in the serum PFOS and perfluorohexane sulfonic acid (PFHxS) levels after 12 months of plasma or blood donations or after observation only. Secondary end points included changes in serum PFAS levels from week 52 to week 64, changes in other PFASs, and changes in complete blood count, biochemistry, thyroid function, and lipid profile from screening to week 52.

**Results:**

A total of 285 firefighters (279 men [97.9%]; mean [SD] age, 53.0 [8.4] years) were enrolled; 95 were randomly assigned to donate plasma, 95 were randomly assigned to donate blood, and 95 were randomly assigned to be observed. The mean level of PFOS at 12 months was significantly reduced by plasma donation (–2.9 ng/mL; 95% CI, –3.6 to –2.3 ng/mL; *P* < .001) and blood donation (–1.1 ng/mL; 95% CI, –1.5 to –0.7 ng/mL; *P* < .001) but was unchanged in the observation group. The mean level of PFHxS was significantly reduced by plasma donation (–1.1 ng/mL; 95% CI, –1.6 to –0.7 ng/mL; *P* < .001), but no significant change was observed in the blood donation or observation groups. Analysis between groups indicated that plasma donation had a larger treatment effect than blood donation, but both were significantly more efficacious than observation in reducing PFAS levels.

**Conclusions and Relevance:**

Plasma and blood donations caused greater reductions in serum PFAS levels than observation alone over a 12-month period. Further research is needed to evaluate the clinical implications of these findings.

**Trial Registration:**

anzctr.org.au Identifier: ACTRN12619000204145

## Key Points

**Question:**

Can levels of perfluoroalkyl and polyfluoroalkyl substances (PFASs) in the blood be reduced by blood or plasma donations?

**Findings:**

In this randomized clinical trial of 285 firefighters, both blood and plasma donations resulted in significantly lower PFAS levels than observation alone. Plasma donation was the most effective intervention, reducing mean serum perfluorooctane sulfonate levels by 2.9 ng/mL compared with a 1.1-ng/mL reduction with blood donation, a significant difference; similar changes were seen with other PFASs.

**Meaning:**

Blood or plasma donations may be used to reduce serum PFAS levels.

## Teaser

This randomized clinical trial examines the effect of blood or plasma donations on serum levels of perfluoroalkyl and polyfluoroalkyl substances among firefighters in Australia.

## Introduction

Perfluoroalkyl and polyfluoroalkyl substances (PFASs) are synthetic compounds used in a wide variety of industrial and consumer products because of their resistance to heat and unique surfactant properties. Uses include nonstick products, such as Teflon, stain- and water-resistant materials, paints, and firefighting foams. Perfluorohexane sulfonate (PFHxS), perfluorooctane sulfonate (PFOS), and perfluorooctanoic acid (PFOA) are commonly detected in human biomonitoring studies.

Perfluoroalkyl and polyfluoroalkyl substances persist in the environment and accumulate in the human body, where they have a prolonged half-life (eg, 4.8 years for PFOS).^1^ The half-life of PFASs in animals is shorter, limiting the utility of animal studies.^2^ The measurement of PFAS chemicals in human populations coupled with their known persistence, bioaccumulation, and toxic effects—particularly at higher levels among workers, such as firefighters,^3^ and in residential communities^4^—has raised concerns about the detrimental effects of PFASs on health. Firefighters have historically been exposed to firefighting foams that contain high levels of various PFASs; previous studies have found that firefighters have higher PFAS levels in their blood, particularly of PFOS and PFHxS, than the general population.^5^

Environmental and health agencies, including the European Chemicals Agency,^6^ the US Agency for Toxic Substances and Disease Registry,^7^ the US Environmental Protection Agency,^8^ and the Australian Government Department of Health,^9^ have identified that PFAS exposure has been associated with adverse health effects. These effects include low fetal weight,^10^ impaired immune response,^11^ thyroid function abnormalities,^12^ obesity,^13^ increased lipid levels,^14,15^ liver function alterations,^16^ and, potentially, an increased risk of some malignant neoplasms.^17^ These associations have been disputed by other authors,^18^ and the exact threshold at which these risks may increase remains unknown. Nonetheless, several long carbon chain PFAS chemicals are considered potentially carcinogenic^19^ as identified by the International Agency for Research on Cancer, who have classified PFOA as a group 2B (possible) carcinogen for kidney and testicular cancers.^20^

Perfluoroalkyl and polyfluoroalkyl substances bind to serum proteins in the blood,^21^ so removal of any blood containing these proteins may, over time, reduce the levels of PFASs in the blood. Observational studies have found lower levels of PFASs in patients undergoing regular venesection.^22^ A small pilot study of 1 family suggested that regular venesection may reduce PFAS levels in the blood.^23^ Premenopausal women have lower PFAS levels than men,^24^ perhaps from depuration of PFASs with regular menstruation.^25^ Perfluoroalkyl and polyfluoroalkyl substances are measured in serum or plasma,^26^ and, given that plasma can be safely removed more frequently than whole blood, donating plasma may be a more effective way to reduce PFAS levels.

To our knowledge, the effect of plasma or blood donations on blood PFAS levels has not previously been studied in a randomized clinical trial. We report the results of our randomized clinical trial examining the effects of 12 months of regular plasma or blood donations on PFOS and PFHxS levels in a cohort of Australian firefighters.

## Methods

### Study Design and Participants

This is an open-label randomized clinical trial of selected Fire Rescue Victoria staff or contractors. We prescreened participants via telephone and email before collecting written informed consent and questionnaires and before obtaining blood samples at a single general practitioner practice. Blood and plasma donations were collected at registered blood networks or at the same general practitioner practice. This trial was approved by the Macquarie University Human Research Ethics Committee. This report follows the Consolidated Standards of Reporting Trials (CONSORT) reporting guideline for randomized studies.^27^

Study participants were current or former Fire Rescue Victoria staff or contractors with serum PFOS levels of 5 ng/mL or more who were eligible to donate blood, had not donated blood in the 3 months prior to randomization, and were able and willing to provide written informed consent. Participants with planned extended leave (eg, >6 weeks) were excluded. Full eligibility criteria are provided in the previously published protocol.^28^

This study was conducted according to International Council for Harmonisation Guidelines for Good Clinical Practice. The trial is registered with the Australian New Zealand Clinical Trials Registry (ACTRN12619000204145; trial protocol in Supplement 1).

### Randomization

We randomly assigned eligible participants to donate plasma every 6 weeks for 12 months, to donate whole blood every 12 weeks for 12 months, or to be observed only. Covariate-adaptive randomization^29^ was used to balance participants’ sex and baseline serum PFAS levels, stratified by quartile, between the 3 groups. Randomization was computer generated and conducted centrally by 1 of the investigators (M.K.F.) who was not involved in the data collection or intervention.

### Procedures

Participants had serum PFAS levels measured at screening, baseline (week 0), week 52, and week 64. Participants randomly assigned to donate plasma gave plasma in amounts up to 800 mL every 6 weeks for a total of up to 9 plasma donations. Participants randomly assigned to donate whole blood gave approximately 470 mL of blood every 12 weeks for a total of up to 5 donations. In both the blood donation and plasma donation groups, the first donation was shortly after the baseline PFAS blood test, and the final donation was scheduled for week 48, allowing 4 weeks between the final donation and the week 52 PFAS blood test. No participants donated between the week 52 PFAS test and the week 64 PFAS test to allow for the evaluation of any changes after donations ceased.

Full blood count, biochemistry, thyroid function, and lipid profile were also assessed at screening and at week 52. Adverse events were reported to the clinical project manager and graded by the principal investigator (R.G.) using the National Cancer Institute Common Terminology Criteria for Adverse Events, version 4.03.^30^ All end point data were verified by an external data monitor prior to analysis.

### Outcomes

The coprimary end points were a change in serum PFOS and PFHxS levels after 12 months of plasma or blood donations compared with baseline levels and with the observation group. Secondary end points included changes in serum PFAS (PFOS or PFHxS) levels in each group from week 52 to week 64; changes in serum levels of 26 other PFAS chemicals (eAppendix in Supplement 2) from baseline to week 52 and from week 52 to week 64; and changes in full blood count, biochemistry, thyroid function, and lipid profile results from screening to week 52.

### Statistical Analysis

Analyses are based on intention to treat with G*Power, version 3.1.9.4,^31^ used for a priori power analyses. As reported in the trial protocol, power analyses treated a 25% reduction in serum PFOS and PFHxS levels as potentially clinically significant. Each group required 94 participants to have 90% power to detect this change within each group, corresponding to a small standardized mean difference (Cohen *d* ≥ 0.31), and 105 participants per group to have 90% power to detect a conventional small effect size difference (partial η^2^ = 0.01) between the intervention groups with a 2-sided α of .05. The actual sample size of 95 participants per group achieves 87% power to detect this effect size between groups and 90% power to detect partial η^2^ = 0.011. To further compare the effect of plasma donation and whole blood donation to that of observation only, with a Bonferroni correction for multiple testing to minimize type II error for the planned post hoc contrasts (2-sided α = .017), the sample of 95 participants per group provides 75% power to detect the same small effect size (partial η^2^ = 0.01 and 90% power to detect partial η^2^ = 0.014).

Primary analyses were planned a priori to examine the mean change within each group from baseline to week 52 for blood serum levels of both PFOS and PFHxS as well as the mean differences between treatment groups for both chemicals at week 52, controlling for their baseline levels. The a priori statistical plan also included several secondary end points, including examining differences in 26 other PFAS chemicals, lipid profiles (total cholesterol, low-density lipoprotein cholesterol, high-density lipoprotein cholesterol, and triglycerides), thyroid function (thyroid-stimulating hormone, unbound T4, and T3), liver function, and kidney function at week 52, controlling for their results at screening. Per-protocol analyses and descriptive analyses of treatment effects stratified by PFOS and PFHxS quartiles were post hoc.

Full details of the statistical methods, including sensitivity analyses, are reported in the eMethods in Supplement 2. Outliers more than 3 SDs from the mean were winsorized to 3 SDs, and bootstrapping was used to account for the remaining nonnormality. The analyses were conducted in a general linear model framework to facilitate robust sensitivity analyses.

We applied a studywide false discovery rate for all *P* values interpreted in the secondary end point analyses (false discovery rate–corrected *P* < .20) to control for multiple comparisons and err on the side of discovery, given that, to our knowledge, this is the first clinical trial to examine the efficacy of blood and plasma donations in lowering serum PFAS levels and correlated physical health indicators.^32^ Results are reported based on the adjusted *P* values, noting that the a priori analytical plan specified that these analyses would focus on effect sizes over statistical significance. All analyses were conducted in SPSS, version 27 (IBM Corp).

## Results

**Figure 1.**
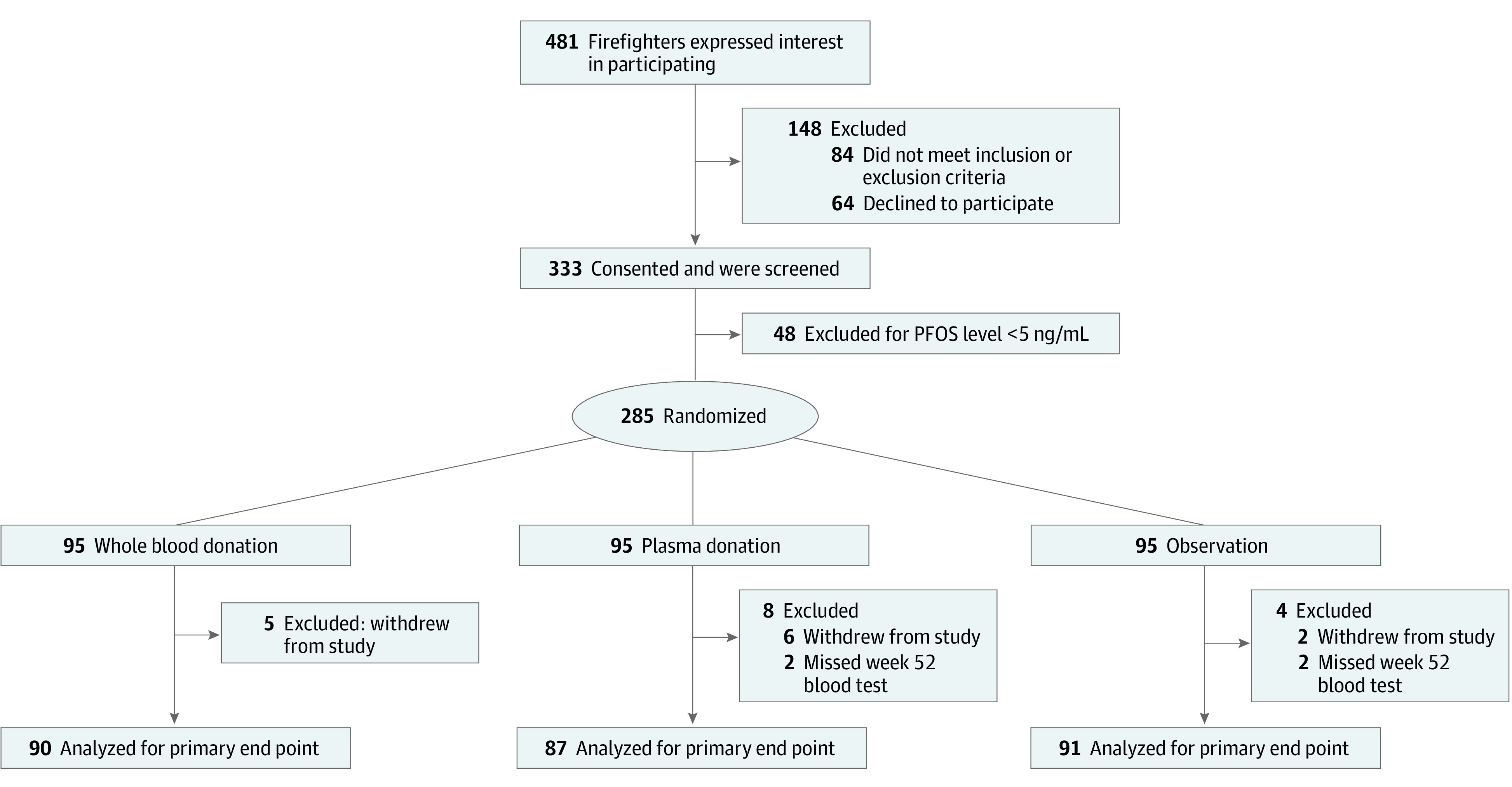
Study Flow Diagram PFOS indicates perfluorooctane sulfonate.

Participant enrollment took place from May 23 to August 23, 2019. A total of 481 Fire Rescue Victoria staff or contractors expressed interest in the study; 333 participants were screened and consented, and 285 met the eligibility criterion of a serum PFOS level of 5 ng/mL or more (Figure 1). Most participants (279 [97.9%]) were male, with a mean (SD) age of 53.0 (8.4) years (Table). Regression toward the mean was evident for serum PFAS levels from screening to baseline, particularly for PFOS, owing to the threshold applied for study eligibility. Participants in the intervention groups completed a mean number of 6.4 (range, 0-9) plasma donations or 4.3 (range, 0-5) blood donations. The participant retention rate was 93.7% (n = 267) for the duration of the study.

**Table.** Screening and Baseline Characteristics of the Intention-to-Treat Population

Variable	Observation group (n = 95)	Blood removal group (n = 95)	Plasma removal group (n = 95)	Total (N = 285)
Age, mean (SD), y	54.3 (7.9)	51.3 (8.4)	53.3 (8.6)	53.0 (8.4) [range, 32-77]
Sex, No. (%)				
Female	2 (2.1)	2 (2.1)	2 (2.1)	6 (2.1)
Male	93 (97.9)	93 (97.9)	93 (97.9)	279 (97.()
Country of birth (Australia), No. (%)	84 (88.4)	87 (91.6)	90 (94.7)	261 (91.6)
Duration of exposure to AFFF, mean (SD), y	23.3 (10.3)	20.4 (9.7)	22.0 (9.4)	21.9 (9.8) [range, 2-46]
History of blood donation, No. (%)	67 (70.5)	57 (60.0)	64 (67.4)	188 (66.0)
BMI, mean (SD)	28.0 (3.4)	27.9 (4.0)	27.9 (3.4)	27.9 (3.6) [range, 19.9-44.6]
PFOS levels^a^				
Screening, mean (SD), ng/mL	12.5 (6.8)	12.4 (9.5)	14.2 (17.9)	13.0 (12.3) [range, 5-170]
Baseline, mean (SD), ng/mL	10.7 (5.9)	10.9 (8.3)	11.7 (20.1)	11.1 (12.9) [range, 2-190]
PFHxS levels^a^				
Screening, mean (SD), ng/mL	4.5 (6.0)	4.3 (6.1)	5.9 (12.9)	4.9 (8.9) [range, 0-120]
Baseline, mean (SD), ng/mL	3.9 (5.7)	3.6 (5.0)	5.2 (15.0)	4.2 (9.7) [range, 0-140]

Abbreviations: AFFF, aqueous film forming foam; BMI, body mass index (calculated as weight in kilograms divided by height in meters squared); PFHxS, perfluorohexane sulfonic acid; PFOS, perfluorooctane sulfonate.

^a^ PFOS and PFHxS values include outliers in the observed raw values reported here; outliers more than 3 SDs from the mean were winsorized to 3 SDs for all other analyses.

### Serum PFOS Levels

**Figure 2.**
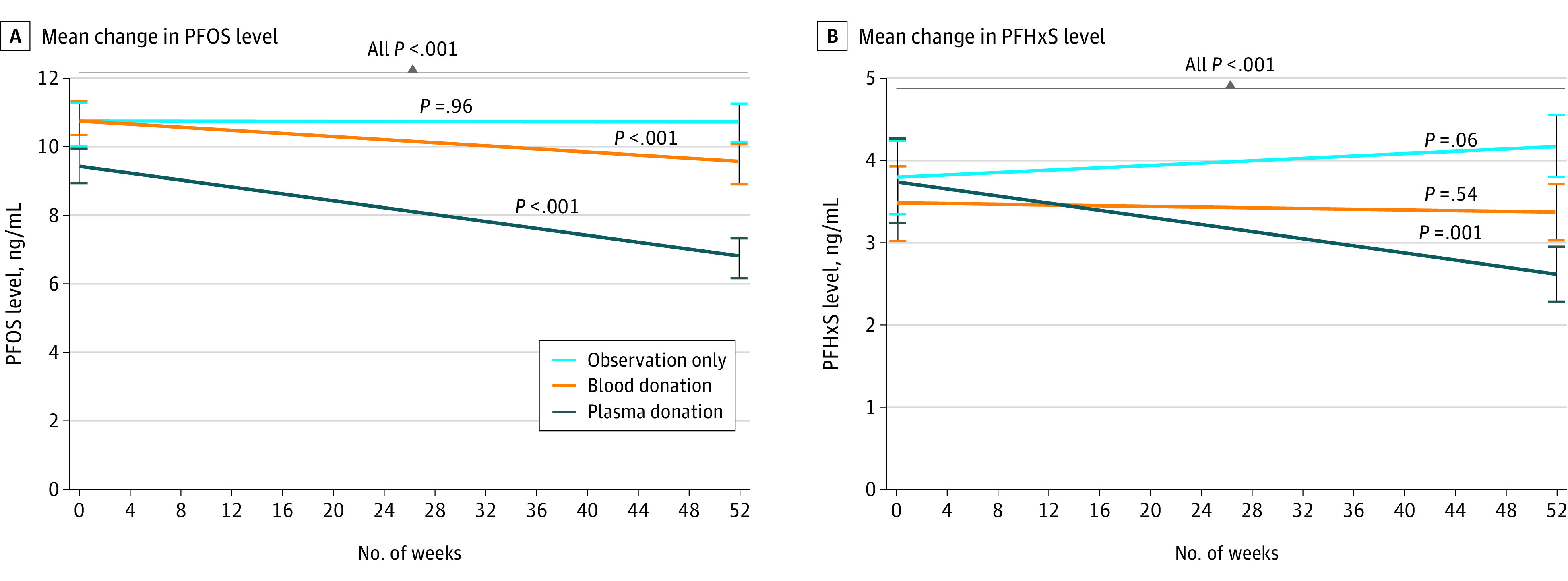
Mean Change in Perfluoroalkyl and Polyfluoroalkyl Substances From Baseline to Week 52 A, Mean change in perfluorooctane sulfonate (PFOS) level from baseline to week 52. B, Mean change in perfluorohexane sulfonic acid (PFHxS) level from baseline to week 52. Error bars indicate the SEM. Change in the observation group was not significant for either outcome (*P* = .96 for PFOS and *P* = .06 for PFHxS); change in the blood donation group was significant for PFOS (*P* < .001) but not for PFHxS (*P* = .54); and change in the plasma donation group was significant for both outcomes (all *P* < .001).

From baseline to week 52 in the plasma donation group, mean PFOS levels were significantly reduced (–2.9 ng/mL; 95% CI, –3.6 to –2.3 ng/mL; *P* < .001) (Figure 2A). For the blood donation group, mean PFOS levels were also significantly reduced (–1.1 ng/mL; 95% CI, –1.5 to –0.7 ng/mL; *P* < .001). By contrast, for the observation group, serum PFOS levels at week 52 were not significantly different from baseline, with a mean decrease of 0.01 ng/mL (95% CI, −0.5 to 0.5 ng/mL; *P* = .96).

Mean PFOS levels were reduced significantly more by both plasma donation (–3.1 ng/mL; 95% CI, −3.8 to −2.4 ng/mL; *P* < .001) (Figure 2A) and blood donation (–1.1 ng/mL; 95% CI, −1.7 to −0.5 ng/mL; *P* < .001) compared with the observation group. Participants who donated plasma had mean PFOS levels that were 2.0 ng/mL lower (95% CI, −2.6 to −1.3 ng/mL; *P* < .001) than PFOS levels among those who donated blood.

### Serum PFHxS Levels

In the plasma donation group, mean PFHxS levels were significantly reduced (–1.1 ng/mL; 95% CI, –1.6 to –0.7 ng/mL; *P* < .001) (Figure 2B). In both the observation and blood donation groups, mean PFHxS levels were not significantly different from baseline, increasing 0.4 ng/mL (95% CI, −0.01 to 0.7 ng/mL; *P* = .06) in the observation group and decreasing 0.1 ng/mL (95% CI, −0.4 to 0.2 ng/mL; *P* = .54) in the blood donation group.

Compared with the observation group, mean PFHxS levels were reduced significantly more by both plasma donation (–1.5 ng/mL; 95% CI, −1.9 to −1.1 ng/mL; *P* < .001) and blood donation (–0.6 ng/mL; 95% CI, −0.9 to −0.2 ng/mL; *P* = .001). Participants who donated plasma had mean PFHxS levels that were 0.9 ng/mL (95% CI, −1.3 to −0.6 ng/mL; *P* < .001) lower than mean PFHxS levels in those who donated blood. Key results from sensitivity analyses for the primary end points are reported in the eMethods in Supplement 2.

### Secondary PFAS End Point Levels

**Figure 3.**
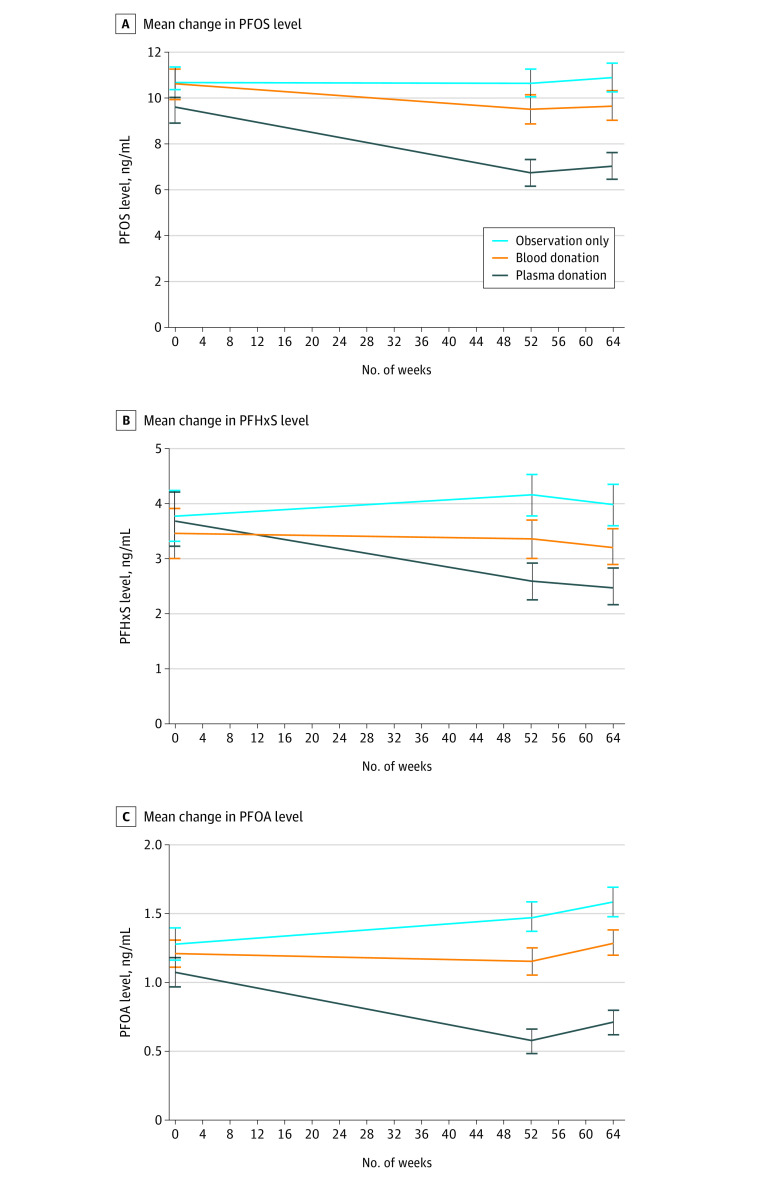
Observed Mean Change in Perfluoroalkyl and Polyfluoroalkyl Substances, Including the Follow-up Period A, Mean change in perfluorooctane sulfonate (PFOS) level, including the follow-up period. B, Mean change in perfluorohexane sulfonic acid (PFHxS) level, including the follow-up period. C, Mean change in perfluorooctanoic acid (PFOA) level, including the follow-up period. Error bars indicate the SEM.

Secondary end points included 26 additional PFASs, but only PFOA had sufficient data for analysis; nearly all (range, 93.0% [265] to 100%) participants had undetectable levels of the other 25 PFASs at baseline. In the plasma donation group, mean PFOA levels were significantly reduced (–0.5 ng/mL; 95% CI, –0.7 to –0.3 ng/mL; *P* = .001) (Figure 3C). In the blood donation group, mean PFOA levels were not significantly different from baseline at week 52, with a reduction of 0.1 ng/mL (95% CI, −0.2 to 0.1 ng/mL; *P* = .63). In the observation group, mean PFOA levels at week 52 had a small but statistically significant increase of 0.2 ng/mL from baseline (95% CI, 0.1-0.3 ng/mL; *P* = .02).

Compared with the observation group, mean serum PFOA levels were reduced significantly more in both the plasma donation group (–0.8 ng/mL; 95% CI, −0.9 to −0.6 ng/mL; *P* = .001) and the blood donation group (–0.3 ng/mL; 95% CI, −0.4 to −0.1 ng/mL; *P* = .007) from baseline to week 52. Participants who donated plasma had mean PFOA levels that were 0.5 ng/mL (95% CI, −0.7 to −0.3 ng/mL; *P* = .001) lower than PFOA levels among those who donated blood.

Mean (SD) PFOA levels were low at baseline (1.2 [1.1] ng/mL) and close to the detection limit of 1 ng/mL across the entire cohort; sensitivity analyses recoding all serum PFOA levels below the limits of reporting at the upper bound of the threshold (<1 ng/mL recoded to 1 ng/mL) showed a smaller mean reduction due to plasma donation from baseline to week 52 (–0.2 ng/mL; 95% CI, –0.4 to –0.1 ng/mL; *P* = .003) and an insignificant increase in the observation group (0.1 ng/mL; 95% CI, 0.0-0.2 ng/mL; *P* = .22). The differences between groups were correspondingly smaller but remained statistically significant even after applying the false discovery rate.

### Other Secondary End Points

Treatment differences between study groups were maintained from week 52 to week 64 for PFOS, PFHxS, and PFOA levels (Figure 3). Across the entire study cohort, however, a slight increase was observed in serum PFOS levels (0.2 ng/mL; 95% CI, 0.1-0.3 ng/mL; *P* = .001) and serum PFOA levels (0.1 ng/mL; 95% CI, 0.1-0.2 ng/mL; *P* = .006), with a slight decrease in PFHxS levels (−0.1 ng/mL; 95% CI, −0.2 to −0.1 ng/mL; *P* = .004).

Hemoglobin levels were reduced more in the blood donation group than in the plasma donation group (−0.51 g/dL; 95% CI, −0.72 to 0.29 g/dL [to convert to grams per liter, multipy by 10.0]; *P* = .001) or the observation group (−0.45 g/dL; 95% CI, −0.64 to −0.25 g/dL; *P* = .001), but otherwise no significant differences were observed for lipid profile, thyroid, liver, or kidney function test results between groups at week 52 after controlling for baseline levels (eResults and eFigures 1-3 in Supplement 2).

Adverse events were more frequent in the plasma donation group (eTable in Supplement 2). A total of 13 (4.6%) participants withdrew from the study, and 268 (94.0%) had complete data for baseline and week 52 PFAS levels; their results are included in the intention-to-treat analysis regardless of how many donations were completed. This intention-to-treat analysis provides a more conservative view of the effect of the interventions because not every participant was able to complete the study as planned. Per-protocol analyses of the subsample of participants with data at week 52 who completed all blood donations (n = 68) or all plasma donations (n = 47) showed similar results with slightly larger effect sizes (eFigure 4 in Supplement 2).

**Figure 4.**
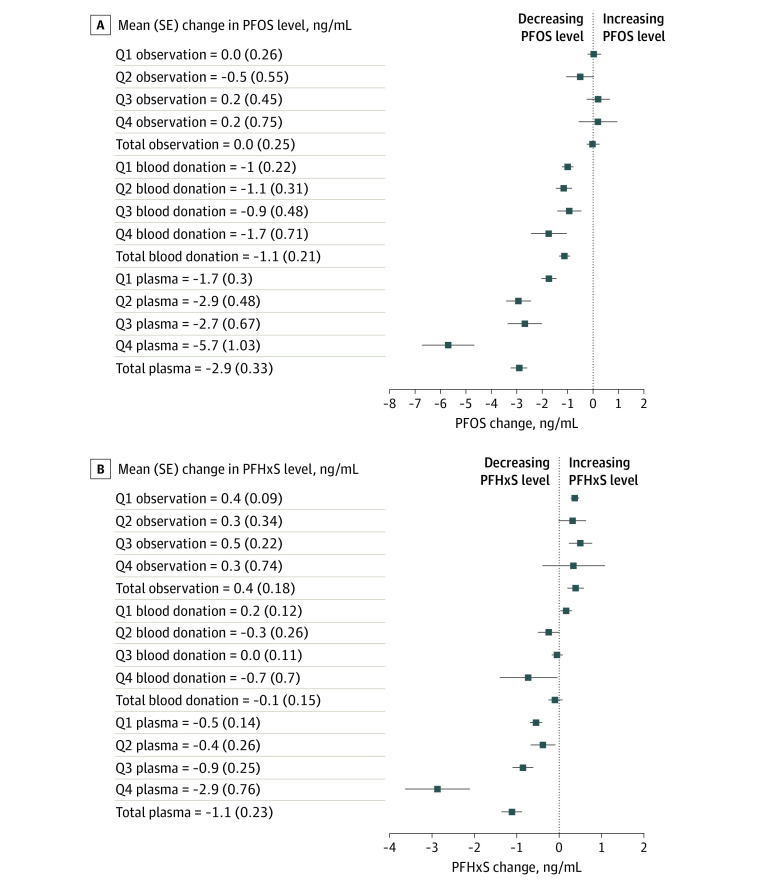
Treatment Effects Stratified by Quartiles at Baseline A, Mean (SE) change in perfluorooctane sulfonate (PFOS) level from baseline to week 52. B, Mean (SE) change in perfluorohexane sulfonic acid (PFHxS) level from baseline to week 52. Q indicates quartile.

A post hoc descriptive analysis of treatment effects stratified by PFOS and PFHxS quartiles at baseline indicated a potential pattern of larger treatment effects for participants with serum levels in the top quartile, particularly for plasma donation. This difference was not apparent in the observation group (Figure 4).

## Discussion

To our knowledge, this is the first randomized clinical trial to systematically quantify whether plasma or blood removal is an effective strategy for reducing serum PFAS levels. Plasma donations resulted in a more substantial decrease in serum PFAS levels than blood donations, and both treatments were more effective than observation alone. This difference may arise because participants in the plasma group were able to donate every 6 weeks rather than every 12 weeks for whole blood. Each plasma donation can amount to as much as 800 mL compared with 470 mL for whole blood; the increased volume may contribute to the faster reduction in serum PFAS levels found in the plasma donation group. In addition, plasma donation may be more efficient at reducing the body’s burden of PFASs because serum PFAS levels are approximately 2 times higher than blood PFAS levels.^26^ On the other hand, plasma donation is more complex, and adherence to the protocol was lower for this group; the mean number of plasma donations was 6.4 of the 9 planned compared with 4.3 of the 5 planned whole blood donations during the study period. Future research should investigate the role of the number, frequency, and volume of each donation to elucidate these likely mechanisms of treatment change.

Mean serum PFOS, PFHxS, and PFOA levels at baseline were lower in this study than the mean levels observed in another cohort of Australian firefighters.^5^ This difference may reflect a natural decrease in PFAS levels over time, as has been described in American Red Cross blood donors,^33^ along with efforts to reduce PFAS exposure among firefighters. The mean PFAS levels found in our study population were similar to those reported for the general Australian population.^5^

Blood donors who have elevated serum PFAS levels are not excluded from donating blood. Perfluoroalkyl and polyfluoroalkyl substances are ubiquitous, and no threshold has been identified that poses an increased risk to recipients of donated blood components. Our study does not inform this risk, but blood authorities should continue to monitor the evidence on the possible health effects of PFASs and consider the possible implications of elevated PFAS levels in blood donors.

We did not find a decrease in serum PFAS levels among participants assigned to the observation group. We observed a small but statistically significant increase in PFOA levels over the 52 weeks among participants assigned to the observation group, but serum PFOS and PFHxS levels were unchanged. The half-lives for PFOS, PFHxS, and PFOA have previously been reported as 4.8, 7.3, and 3.5 years, respectively,^1^ so during a 12-month period, we would have expected PFAS levels to decrease by approximately 10%. In our cohort, the baseline levels were substantially lower than those in the previous study,^5^ which may explain the lower-than-expected changes in PFAS levels in the control group. In addition, the observed changes may be due to ongoing environmental exposure or redistribution between body compartments (eg, moving from liver to plasma), but the limit of detection for the PFAS assays was 1 ng/mL, so any changes close to this threshold need to be interpreted with caution.

### Limitations

This study has some limitations. Serum PFAS levels were measured at screening, baseline, week 52, and week 64 but were not assessed during the intervention (ie, between baseline and week 52), so we are not able to comment further on the kinetics of PFAS clearance. However, the treatment effects were maintained during the 12-week follow-up period from week 52 to week 64. Although these study data provide evidence for sustained efficacy of plasma and blood donations to reduce serum PFAS levels, extended follow-up of the cohort would be useful to assess changes in PFAS serum levels over time.

Although elevated PFAS levels have previously been shown to be associated with hyperlipidemia,^15^ elevated liver function test results,^16^ and thyroxine levels,^12^ we did not see any significant change in lipid levels or other clinical blood test results, with the exception of lower hemoglobin levels from blood donations, as a result of these interventions. This outcome is perhaps not surprising in a relatively small heterogenous cohort. Larger studies examining these clinical end points should be performed.

As for PFOA levels in the observation group, minor changes were noted in PFAS levels during this follow-up period; PFOS and PFOA levels increased slightly, whereas PFHxS levels decreased slightly. It is not clear whether these postintervention changes reflect ongoing exposures, shifts from body tissue stores of PFAS into plasma, or variation in the PFAS assay. Further follow-up of this study cohort would be helpful to address this question as well as to explore the potential mechanisms for treatment effects from baseline to week 52.

In 2016, the Human Biomonitoring Commission of the German Environment Agency determined human biomonitoring (HBM)–I plasma levels of 5 ng/mL for PFOS and 2 ng/mL for PFOA, below which no adverse health effects are expected.^34^ More recently, the Human Biomonitoring Commission derived HBM-II plasma thresholds for the general population, excluding women of childbearing age, of 20 ng/mL for PFOS and 10 ng/mL for PFOA, which, when exceeded, may lead to health impairments.^35,36^ Given the long half-life of PFASs both for bioelimination and environmental persistence, ongoing exposures will persist for decades. Regulations to reduce widespread PFAS exposure are needed, but an intervention that can reduce PFAS levels in exposed populations would be a useful adjunct.

## Conclusions

This randomized clinical trial showed that regular blood or plasma donations result in a significant reduction in serum PFAS levels for participants with a baseline PFOS level of 5 ng/mL or more; plasma donations reduced levels more quickly than blood donations. Blood and plasma removal are relatively straightforward procedures, and, provided they are performed under medical supervision, the risks to the patient are minimal. Further research is warranted to investigate the clinical effects of reducing PFAS levels and to better define the cohorts who would benefit most from these interventions.

## Notes

Supplement 1.
Trial Protocol

Supplement 2.
eMethods.

eResults.
**eFigure 1.** Association Between Screening and Week 52 Levels of TSH **eFigure 2.** Association Between Screening and Week 52 Levels of ALT **eFigure 3.** Association Between Screening and Week 52 Levels of Platelets **eTable.** Adverse Events **eFigure 4.** Per-Protocol Analysis
eAppendix.

Supplement 3.
Data Sharing Statement
