# Supplementary material for: Effect of Plasma and Blood Donations on Levels of Perfluoroalkyl and Polyfluoroalkyl Substances in Firefighters in Australia: A Randomized Clinical Trial
Source: JAMA Netw Open. 2022 Apr 8;5(4):e226257. doi: 10.1001/jamanetworkopen.2022.6257 (PMC8994130; doi:10.1001/jamanetworkopen.2022.6257)
Supplement: Supplement 1. — Trial Protocol [file jamanetwopen-e226257-s001.pdf]

Faculty of Medicine and Health Sciences
Department of Clinical Medicine
75 Talavera Rd, Macquarie University,
SYDNEY, NSW 2109, Australia

MACQUARIE University SYDNEY·AUSTRALIA

A randomised controlled trial examining the effect of blood and plasma donation on serum per- and poly-fluoroalkyl substances (PFAS) levels in Metropolitan Fire Brigade staff

Investigators team:

1. Principal Investigator:
   Dr Robin Gasiorowski MA (Cantab) MBBS, PhD, FRACP, FRCPA
   Consultant Haematologist, MQ Health & Concord Hospital, Senior Lecturer, Macquarie University & University of Sydney
   Faculty of Medicine and Health Sciences
   Macquarie University, Sydney
   2 Technology Place NSW 2109
   T: +61 (2) 9812 2981
   E: robin.gasiorowski@mq.edu.au

2. Chief Investigator:
   Prof Mark Taylor, BSc (Hons), PhD, FRSN
   Dept of Environmental Sciences
   Faculty of Science & Engineering
   Macquarie University, Sydney
   NSW, 2109
   T: +61 (2) 9850 4221
   E: mark.taylor@mq.edu.au

3. Chief Investigator:
   Dr Miriam (Miri) Forbes BA Psych (Hons), PhD
   Research Fellow at Centre for Emotional Health
   Department of Psychology
   4 First Walk
   Macquarie University, NSW, 2109
   T: +61 (2) 9850 9436
   E: miri.forbes@mq.edu.au

4. Chief Investigator:
   Associate Prof Merrole Cole-Sinclair
   Head, Laboratory Haematology, Pathology
   St Vincent's Hospital
   41 Victoria Parade
   Fitzroy VIC 3065
   T: +61 3 9231 3996
   E: merrole.cole-sinclair@svha.org.au

Protocol writing team:
Dr Robin Gasiorowski, Macquarie University, NSW
Dr Miriam Forbes, Macquarie University, NSW
Prof Mark Taylor, Macquarie University, NSW
Associate Prof Merrole Cole-Sinclair, St Vincent's Hospital, Victoria
Dr Yordanka Krastev, Macquarie University, NSW
Dr Brenton Hamdorf, Macquarie University, NSW
Mr Barry Lewis, Metropolitan Fire Brigade (MFB), Victoria
Mr Gabriel Silver, Macquarie University, NSW

Acknowledgements:
Prof Howard Gurney, Director Clinical Trials Unit, Faculty of Medicine and Health Sciences, Macquarie University;
Mr Mick Tisbury, Metropolitan Fire Brigade (MFB), Victoria

Please note: The Metropolitan Fire Brigade became part of the newly formed Fire Rescue Victoria on 1 July 2020, as part of the Fire Services Reform program for Victoria (https://www.vic.gov.au/fire-services-reform) that combined the Country Fire Authority with the Melbourne based Metropolitan Fire Brigade into a single entity.

Synopsis

The aim of the study is to assess the effect of 12 months of blood or plasma donation on serum PFAS levels in a cohort of Metropolitan Fire Brigade (MFB) staff.

Hypotheses

1. Both active interventions— (1) whole blood donation every 12 weeks and (2) plasma donation every 6 weeks—will result in a significant reduction in the serum PFAS levels in the blood of MFB staff.
2. Regular plasma donation will reduce serum PFAS levels in the blood of MFB staff at a faster rate than the whole blood donation; and both whole blood and plasma donation will reduce serum PFAS levels at a faster rate than the clearance rates of MFB staff in the observation-only group.

Co-Primary Endpoints

To identify whether there is a significant difference in serum 1) PFOS and 2) PFHxS levels after 12 months of whole blood or plasma donation within and between groups.

Study design

A randomized, controlled, Phase II study of MFB staff with previous occupational exposure to PFAS.

Study procedures

A total of 315 participants (105 per group) will evenly randomised between (1) whole blood donation, (2) plasma donation and (3) observation (control)only groups. Randomisation will be stratified by sex and baseline PFOS levels to ensure an even distribution of participants across all groups. The study will run for a total of 18 months (approximately 3 months screening and randomisation, 12 months intervention, and 3 month follow up).

Participants in the whole blood donation group will donate five times during the study. Serum PFAS levels will be tested 4 times. Biochemistry tests will be collected twice during the study.

Participants in the plasma donation group will donate nine times. Serum PFAS levels will be tested four times. Biochemistry tests will be collected twice during the study.

Participants in the observation-only group will have serum PFAS levels tested four times. Biochemistry tests will be collected twice during the study.

If separate optional consent is given, a biobanking sample shall be collected once, at Week 52. This applies to all three groups.

Significance

If blood or plasma donation significantly reduces serum PFAS levels, this may inform strategies to reduce the health risks associated with occupational PFAS exposure.

Study sponsorship

This is an investigator-initiated clinical study conducted by Macquarie University and funded by a grant from the Metropolitan Fire and Emergency Services Board.

Terminology and abbreviations

PFAS – Per- and polyfluoroalkyl substances

PFOS – Perfluorooctane sulfonic acid

PFHxS – Perfluorohexane sulfonic acid

PFOA – Perfluorooctanoic acid

MFB – Metropolitan Fire Brigade.

MFB staff – includes any person who is currently or previously employed or contracted by MFB or its subsidiary FES (Fire Equipment Services) and has had occupational exposure to PFAS as a current or former firefighter, workshop staff or FES staff member.

Screening – period when potential study participants are consented for the study and initial blood tests are performed.

Baseline – Day 0, Week 0 of the study.

Follow up period – 3 months after the end of the intervention.

Ethics approval – ethics approval obtained by NHMRC registered Macquarie University HREC.

Advisory panel – a panel of clinicians and independent PFAS experts.

Biobank – A biobank is a stored collection of human biological samples (e.g. tissue, blood or bone) and/or their products (e.g. DNA, associated with personal information).
Biobanks are an important resource for medical researchers to improve the understanding of diseases and to help find better ways to prevent or treat them.

Contents

Synopsis 3

- Hypotheses 3
- Endpoint 3
- Study design 3
- Study procedures 3
- Significance 3
- Study sponsorship 3

Terminology and abbreviations 4

Background and Rationale 6

Hypothesis 7

- Co-Primary Endpoints 7
- Secondary Endpoints 7

Study Design and purpose: 7

Inclusion/ Exclusion Criteria: 10

Intervention 11

- Participant visits and time commitment: 11

Medical oversight of the participants throughout the study: 12

Study procedures: 12

Blood sample collection for biobanking 12

Statistical considerations 12

- Significance 13

Informed Consent 13

Confidentiality 14

Publication policy 14

Appendix 1: Baseline Questionnaire 16

Appendix 2: Schedule of assessments 21

Appendix 3: Blood samples collection, storage and transport 22

Appendix 4 – PFAS Chemicals Tested 23

Appendix 5 - Advisory panel 23

Appendix 6 – Briefing Document for GP's for distribution of PFAS test results 24

References 25

Background and Rationale

Per- and polyfluoroalkyl substances (PFAS) are a diverse group of compounds resistant to heat, water, and oil. Perfluorohexane sulfonate (PFHxS), perfluoroctane sulfonate (PFOS) and perfluorooctanoic acid (PFOA) are the most prominent and commonly detected PFAS. For many years, they have been used in hundreds of industrial applications and consumer products such as carpeting, apparel, upholstery, food paper wrappings, fire-fighting foams and metal coatings. According to the US EPA (USEPA 2018), PFAS have been found at very low levels both in the environment and in the blood samples of the general U.S. population, because they tend to persist long-term in the environment and in the body.

In a study conducted by Rotander et al. (2015) on 149 firefighters working with aqueous film forming foam (AFFF) training facilities in Australia, it was found that the concentrations of PFOS and PFHxS (the key PFAS contaminants) in the firefighter blood were associated with the number of years of exposure they had to AFFF. This is of some concern given the increasing literature evidence that PFAS could have adverse effects on health. For instance, the Centers for Disease Control and Prevention (CDC) suggests that PFAS may affect the developing foetus and child, decrease fertility, increase cholesterol, affect the immune system and increase cancer risk (ATSDR 2018). Elevated PFAS levels have also been reported to be associated with increased liver enzyme levels by Gleason et al. 2015 and increased cholesterol levels by Steenland et al. 2009.

Overall, however, the exact health effects of elevated serum PFAS are not clear. Some studies have reported an association between PFAS levels and thyroid disease (Knox et al. 2011), but the effect has not been consistent across all studies. A number of studies have assessed the possible link between PFAS levels and cancer risk, but the findings have been mixed without a conclusive causal relationship established at this stage (Chang et al. 2011).

PFAS are known to be highly protein bound (Jones et al. 2012) and whilst their exact route of elimination is unclear, they appear to have a prolonged half-life in humans, measuring 8.5 years for PFHxS, 5.4 years for PFOS and 3.8 years for PFOA in one study (Olsen et al. 2007). This compares to a half-life of only days in rodents (Kim et al. 2016), making animal studies on their effects difficult to interpret. Studies have shown that women have lower levels of PFAS, perhaps due to elimination by menstruation (Wong et al. 2014). In addition, previous small studies have suggested that regular phlebotomy may reduce serum PFAS levels (Genius et al. 2014; Lorber et al. 2015) and firefighters who donated blood were found to have lower PFAS levels (Rotander et al. 2015).

Whilst PFAS levels do appear to slowly drop over time once the source of exposure has been eliminated (Olsen et al. 2007), their potential adverse health effects indicate the importance of developing an intervention to bring down high levels at a faster rate. The preliminary evidence suggests that a significant proportion of PFAS resides in the plasma of exposed individuals. Therefore, we hypothesise that regular phlebotomy (i.e. removal of whole blood) and donation of plasma will both be effective in reducing serum PFAS levels. Donation of plasma may be even more effective than whole blood donation, given plasma is likely to contain the majority of total blood PFAS, and plasma donation can occur more frequently than whole blood donation.

Hypothesis

1. Both active interventions— (1) whole blood donation every 12 weeks and (2) plasma donation every 6 weeks—will result in a significant reduction in the serum PFAS levels in the blood of MFB staff.
2. Regular plasma donation will reduce serum PFAS levels in the blood of MFB staff at a faster rate than the whole blood donation; and both whole blood and plasma donation will reduce serum PFAS levels at a faster rate than the clearance rates of MFB staff in the observation-only group.

Co-Primary Endpoints

- To identify whether there is a signficant difference in serum PFOS levels after 12 months of whole blood or plasma donation within and between groups.

- To identify whether there is a signficant difference in serum PFHxS levels after 12 months of whole blood or plasma donation within and between groups.

Secondary Endpoints

1. To identify whether there is a significant change in serum levels of other PFAS chemicals (PFOA, PFBS, PFPeS, PFHpS, PFDS, PFBA, PFPeA, PFHxA, PFHpA, PFNA, PFDA, PFUnDA, PFDoDA, PFTrDA, PFTeDA, 4:2 FTS, 6:2 FTS, 8:2 FTS, 10:2 FTS, FOSA 10, MeFOSA, EtFOSA, MeFOSE, EtFOSE, MeFOSAA, EtFOSAA) after 12 months of whole blood or plasma donation.
2. Changes in serum PFAS (PFOS or PFHxS) levels in each group from post-test to 3-month follow-up.
3. Changes in serum levels of other PFAS chemicals (PFOA, PFBS, PFPeS, PFHpS, PFDS, PFBA, PFPeA, PFHxA, PFHpA, PFNA, PFDA, PFUnDA, PFDoDA, PFTrDA, PFTeDA, 4:2 FTS, 6:2 FTS, 8:2 FTS, 10:2 FTS, FOSA 10, MeFOSA, EtFOSA, MeFOSE, EtFOSE, MeFOSAA, EtFOSAA) in each group from post-test to 3 month follow-up
4. Significant differences in lipid profile (total cholesterol, LDL, HDL and triglycerides) between groups.
5. Significant differences in thyroid function tests (TSH, T4, T3) between groups.
6. Significant differences in liver function tests (bilirubin, ALT, AST, GGT and albumin) between groups.
7. Significant differences in renal function tests (Electrolytes, urea, creatine) between groups.

Study design and purpose

This project will comprise a randomized, controlled, Phase II study of MFB staff with previous occupational exposure to PFAS. The study will investigate whether a simple intervention (whole blood or plasma donation) might have an impact on the elimination or reduction of PFAS from MFB staff's blood. This potential outcome stems from earlier observations (Kato et al., 2011; Toms et al., 2014 and Wong et al., 2014) that menstruating women have lower PFAS levels than men with equivalent exposure. Additionally, preliminary data collected by Rotander et al. (2015) demonstrated that Australian firefighters have elevated PFAS levels (PFOS and PFHxS) in their blood compared to the general population making them a suitable

cohort to test the efficacy of phlebotomy as a possible PFAS treatment. Rotander et al. (2015) also showed an association between lower PFAS levels and blood donation in this group.

Other emerging evidence demonstrating the potential of phlebotomy for PFAS removal in humans comes from the assessment of six single Canadian family members who were subject to intermittent phlebotomy over a 4–5 year period (Genius et al. 2014). This small cohort study indicated that the half-life of PFAS was shorter in the treated group than that in the general population (Genius et al. 2014).

This project is focused on gathering and evaluating data from a discrete occupational cohort – MFB staff who have a history of exposure to per- and polyfluoroalkyl substances (PFAS). Through this study and future studies, the project team seeks to gain insights into the long-term human health effects of exposure to this group of chemicals. The majority of research studies have undertaken cross-sectional analysis of PFAS exposure levels and as a result have not been able to evaluate insights into how quickly PFAS is eliminated (or accumulated). Moreover, there has been no systematic randomised study of any procedure to eliminate PFAS in humans, which is what this project will examine.

There remains significant debate around the issue of long-term detrimental effect due to elevated PFAS exposure (Kirk et al. 2018). Notwithstanding this ongoing debate, the emerging evidence supports the argument that there are a range of adverse health outcomes associated with exposure (Rappazzo 2017; ATSDR 2018), hence the project objective of quantifying the potential of phlebotomy to reduce the concentrations of PFAS chemicals in humans. The identified adverse human health outcomes include:

- **Hepatic effects.** Increases in serum enzymes and decreases in serum bilirubin observed in studies of PFOA, PFOS, and PFHxS are suggestive of liver damage. In addition, the results of epidemiology studies of PFOA, PFOS, PFNA, and PFDeA suggest a link between per- and polyfluoroalkyl exposure and increases in serum lipid levels, particularly total cholesterol and LDL cholesterol.

- **Cardiovascular effects.** There is emerging epidemiological evidence of an association between serum PFOA and PFOS and pregnancy-induced hypertension and/or pre-eclampsia.

- **Endocrine effects.** Epidemiology studies indicate that there is a link between serum PFOA and PFOS and an increased risk of thyroid disease.

- **Immune effects.** Elevated serum PFOA, PFOS, PFHxS, and PFDeA levels have been associated with decreased antibody response to vaccines. A possible link between serum PFOA levels and increased risk of asthma diagnosis has also been identified.

- **Reproductive effects.** Research studies have indicated that there is an association between elevated serum PFOA and PFOS levels and an increased risk of decreased fertility.

- **Developmental effects.** Serum PFOA and PFOS levels is associated with small decreases in birth weight; the decrease in birth weight is <20 g per 1 ng/mL increase in blood PFOA or PFOS level.

- **Kidney and testicular cancer.** Research studies do not clearly show whether PFAS is the cause cancer in people although the International Agency for Research on Cancer has classified PFOA as possibly carcinogenic (IARC 2018). The available research evidence indicates that people exposed to high levels may have increased risk of kidney cancer or testicular cancer.

We will use covariate-adaptive randomisation to balance participants' sex and blood PFAS levels between the three groups.

The whole blood intervention groups will donate blood every 12 weeks while the plasma donation group will be subject to their donation at twice the frequency, i.e. every 6 weeks. The rationale for the differential interventions between the two groups is the limitation on the frequency that whole blood donation can be made. That limit is once every three (3) months. In contrast, plasma donations can be made every six (6) weeks, thus potentially increasing the clearance rate of PFAS.

The study protocol is designed with PFAS tests at the beginning and end of the study. The first tests (at screening and Day 0, Week 0) are designed to establish natural underlying clearance rates of PFAS in the participants. The literature indicates that longer carbon chain PFAS chemicals including the primary target compounds of PFOS and PFHxS have a long half-life in humans and are eliminated slowly (e.g. Zhang et al. 2013).

The third PFAS test (week 52) will assess the impact of the interventions (blood donations) and whether the levels of circulating PFAS have been reduced.

The final test (week 64) will assess whether PFAS levels at the end of study increase or continue to fall as per natural levels established in the study's first two PFAS tests. There is a possibility that the intervention will only facilitate the reduction of circulating PFAS and will not impact PFAS stored in other tissues, which might cause a small rise in circulating PFAS levels.

The study also includes biobanking and biochemistry. Biochemistry samples shall be taken at Screening and Week 52, with an optional biobanking sample also taken at Week 52, if a separate consent is signed by the participant. Biochemistry measures will assess samples for biomarkers of the various health impacts described above and may provide some initial insights in early adverse effects of PFAS exposure at different levels. However, it is predicted that the real value of this data set will accumulate over time and via comparison with future biochemical analyses of the same cohort. Whilst these future activities are not yet supported financially, nor with any definitive study design, it is critical to gather baseline data to support future studies as opportunities emerge.

Participant recruitment

Interested MFB staff will be invited to attend a screening visit where medically qualified clinical study staff will obtain informed consent and conduct the initial screening for the study. Clinical staff will also discuss the trial protocol with participants and answer any questions they may have.

Once participants have consented to take part in the study, they will be invited to complete a baseline questionnaire (shown in Appendix 1).

Blood sample collection:

- A blood sample (8 ml) for assessment of baseline *serum PFAS levels*.
- A blood sample (12ml) for *biochemistry* testing

After completing the above steps and conducting initial PFAS and questionnaire analysis, the research team will determine eligible participants for enrolment into the study based on the following inclusion and exclusion criteria.

Inclusion Criteria:

Potential participants must meet ALL the following inclusion criteria to be eligible for enrolment into the study:

1. Current or former Metropolitan Fire Brigade staff or contractors, with 10 or more years of previous occupational exposure to PFAS or with known elevated PFAS levels (PFOS ≥ 5ng/mL).
2. PFOS levels ≥ 5ng/mL
3. Eligible to donate blood.
4. Not donated blood in the past 3 months prior to randomization.
5. Signed and dated informed consent document indicating that the participant has been informed of all pertinent aspects of the trial prior to enrolment.
6. Willingness and ability to comply with scheduled visits, laboratory tests and other study procedures.

Exclusion Criteria:

Subjects presenting with any of the following will be excluded in the trial:

1. Medical contraindication to blood donation.
2. Other severe acute or chronic medical or psychiatric condition, or laboratory abnormality that may increase the risk associated with study participation or may interfere with the interpretation of study results, and in the judgment of the investigator would make the participant inappropriate for entry into this study.
3. Planned travel or extended leave (e.g. >6 weeks) that would prevent access to blood donation facilities.

The randomisation will be computer generated and will include stratification by sex and baseline PFAS level to ensure an even distribution of participants across all the following three groups

- Group 1: Whole blood donation every 12 weeks
- Group 2: Plasma donation every 6 weeks
- Group 3: Observation only (control group).

A total of 315 participants (105 per group) will be randomized into the study for a 12-month period and 3 months follow up.

Intervention

Participants randomised to standard whole blood donation will donate blood at Blood Service collections centre every 12 weeks. Participants will be required to complete standard Blood Service screening procedures including completion of a confidential health questionnaire and a finger prick blood test to check the haemoglobin level. Once eligibility is confirmed participants will donate a unit of whole blood (approximately 470mls), following standard blood donation procedures. Participants will obtain proof of their blood donations either at the time of donation or at the end of intervention period and book a time for their next donation in 12-weeks.

Participants randomised to standard plasma donation will donate plasma at a Blood Service collections centre every 6 weeks. Participants will be required to complete standard Blood Service screening procedures including completion of a confidential health questionnaire and a finger prick blood test to check the haemoglobin level. Once eligibility is confirmed participants will donate plasma (up to 800mls, depending on height and weight), following standard blood donation procedures. Participants will obtain proof of their plasma donations either at the time of donation or at the end of the intervention period and book a time for their next plasma donation in 6-weeks.

Serum PFAS levels will be checked at baseline, end of the intervention, and at 3 month follow up.

Biobank sample will be collected at Week 52 visit. Participants will sign a separate consent for biobanking blood and DNA, for an initial period of 20 years.

All study procedures and interventions should occur within a timeframe of +/- two weeks of the times specified in the protocol. The study will be conducted for a total of 18 months (approximately 3 months screening and randomisation, 12 months intervention and 3 month follow up).

Participant visits and time commitment

Participants will have a number of visits during the study. The time commitment will depend on the study group they are allocated.

For example:

1. Whole blood donation group – 5 x blood donations (approximately 1hour per donation = 5 hours), 4 x PFAS tests (15min per test =1 hour), 2 x biochemistry (10min each=20min), 1X biobanking sample (10min) = Total estimated time = 6-7hrs

2. Intervention group (plasma donation) – 9 x plasma donations (approximately 1.5 hours per donation = 14 hours) 4 x PFAS tests (15min per test =1 hour), 2 x biochemistry (10min each=20min), 1X biobanking sample (optional)(10min) – Total estimated time = 16-17 hrs

3. Observation only group –2 x biochemistry (10min each=20min), 1X biobanking sample (10min), 4 x PFAS tests (15min per test =1 hour) – Total estimated time = 1-2hrs

Medical oversight of the participants throughout the study

Participants will have data collected after every blood or plasma donation, to ensure that they are feeling well, and that the donation has occurred. Participants will have to confirm the date that they donated blood/ plasma and whether there were any adverse effects from the procedure.

If they report any issues, this will be followed up by a phone call from the study co-ordinator to establish the details. Participants will be directed to their GP/ ED if there are any significant issues. The P.I. Dr Gasiorowski will review and sign off every Adverse Event Report in a timely manner. The Advisory Panel shall review any reports of these adverse events for trends. Any serious advent events, e.g. those requiring hospitalisation, will be reported back to the study sponsor within 24 hours according to GCP, and be reviewed by an Investigator within 24hrs of knowledge.

Study procedures

Aside from visits and donations already described in detail, trained phlebotomists from a nominated pathology company will take blood samples for analysis. These will include:

- Blood/serum for PFAS testing- 1 x 8ml SST
- Blood/serum for biochemical testing – 1 x 8ml SST, 1x4mL EDTA
- Blood for biobanking- 1 x 8-10mL SST and 1x 10mL EDTA

PFAS, biochemistry and biobank blood samples collection, storage and transport are listed in Appendix 3

PFAS chemicals to be tested are listed in Appendix 4.

Blood sample collection for biobanking

An 8-10ml SST and 10mL EDTA vial of blood will be collected for biobanking as this study will provide an excellent opportunity to collect samples from a large and unique occupational cohort of MFB staff. A separate informed consent will be sought from participants for collection of blood samples for biobanking purposes.

Statistical considerations

We would consider a 25% reduction in serum PFOS and PFHxS levels to be significant after 12 months of plasma or whole blood donation. Based on a 25% reduction in the mean serum levels of PFHxS and PFOS, found in 149 Australian firefighters in Rotander *et al*. (2015), and a correlation between the assessments at baseline and 12 months (post-test) of r = .6, this corresponds to a standardised effect size of mean difference $d_z \geq .31$. We would require each group to have 94 participants to have 90% power to detect a 25% reduction in PFOS and PFHxS levels.

In order to test whether plasma donation reduces serum PFOS and PFHxS levels at a faster rate than whole blood donation, we would require each group to have 105 participants at pre-test for 90% power to detect a conventional small effect size (partial eta-squared of .01) difference between the groups from pre-test to post-test.

To further compare the efficacy of plasma donation and whole blood donation to the observation only group (control), a sample of 105 participants per group would provide 80% power to detect the same small effect size (and 90% power to detect partial $\eta^2 = .013$) after correction for multiple testing.

We have planned our analyses based on intention-to-treat and will use multiple imputation to handle missing data. Our power analyses are conservative to account for the possibility of inflated Type II error and indicate a required total sample size of 315 participants (105 per group). The protocol is designed to allow for up to 10% of participants withdrawing from treatment over the life of the project.

Change in each Secondary Endpoint will be deemed substantive and indicate further investigation in future research if change in the endpoint corresponds with at least a small standardized effect size; statistical significance will not be a focus in these analyses, as we will be underpowered to correct for multiple comparisons.

Significance

If whole blood or plasma donation significantly reduces serum PFAS levels, this may inform strategies to reduce the potential health risks associated with occupational PFAS exposure.

Informed Consent

Before study initiation, the protocol and all study related documents will be submitted for review and final approval by the Macquarie University HREC.

Informed consent will meet the requirements of the latest revision of the National Statement on ethical conduct in human research and the Guidelines for Good Clinical Practice (GCP) in Australia. The study will be explained to each potential participant by a trained clinician and the subject must give informed consent by signing and dating the consent form. Informed consent will be obtained before any protocol-required procedures or investigations are performed. The investigator will provide the subject a printed copy of the participant's information sheet and consent form.

All participants will be informed of the aims of the study, the possible adverse events, the procedures and possible hazards to which he/she will be exposed. They will be informed as to the strict confidentiality of their participant data, but that their medical records may be reviewed for clinical research purposes by authorised individuals other than their treating physician. A separate informed consent will be sought for biobanking purposes and future use of the collected samples for research (this will be optional).

It will be emphasised that the participation is voluntary and that the participant is free to refuse further participation in the study whenever he/she wants. Documented informed consent must be obtained for all participants included in the study before they are registered or randomised in the study. This must be done in accordance with the national and local regulatory requirements.

The informed consent procedure must conform to the ICH GCP guidelines. This states that "the written informed consent form should be signed and personally dated by the participant or by the participant's legally acceptable representative".

The participants will not have access to their PFAS blood test results until after the study is completed. This will reduce the impact of any confounding variables. (e.g. MFB staff in the control group doing undisclosed blood donations if they find they have high PFAS levels in the early part of the study, or those with low levels skipping donation appointments). The blood test results will be sent to the GP at the end of the study, accompanied by a letter from the Principal Investigator and a PFAS blood result fact sheet.

Confidentiality

Confidentiality will be maintained at all times. Participants will be assigned a study number and original data will be deidentified. This data will be stored in a secure office and entered into a secure project database that will be password protected. The information will be stored for an indefinite period. All persons who are to have access to the deidentified data shall complete a signed declaration binding them to respect the confidentiality of the information contained therein. This includes access by MFB, any of the investigators, study staff, or other third parties requesting access to such data. A record of all access provided and what was accessed will also be retained.

A master log with Study number-participant identification key shall be kept by the Principle Investigator and the study coordinator electronically to provide the ability to follow-up participants using contact information. Access to this log shall be via secure user name and password on an encrypted laptop. At no time will identifiable data be released.

Paper participant records (includes consents, questionnaires, and biochemistry blood results) are kept in a locked, secure filing cabinet at the home address of the Melbourne based Clinical Project Manager. At the end of the study these shall be archived as per MQ University protocol through Archives and Records Management. E: ask.memory@mq.edu.au; T: +61 2 9850 7362, Ground Floor, The Chancellery, 19 Eastern Road, Macquarie University, NSW 2109, Australia. The electronic files (includes ISF and participant PFAS blood results) are kept in the password protected study iCloud storage file on MQ SharePoint, where they shall also be archived.

Publication policy

To coordinate dissemination of data from this study, a publication committee consisting of several investigators, appropriate clinical study coordination staff, and MFB representation shall be formed. The committee is expected to solicit input and assistance from other investigators and clinical research staff as appropriate. Membership on the committee does not guarantee authorship—the criteria described below should be met for every publication.

Authorship of any publications resulting from this study will be determined on the basis of the Uniform Requirement for Manuscripts Submitted to Biomedical Journals (International Committee of Medical Journal Editors (ICMJE), 2004), which states:

(A) Authorship credit should be based on (1) substantial contributions to conception and design, acquisition of data, or analysis and interpretation of data; (2) drafting the article or revising it critically for important intellectual content; (3) final approval of the version to be published. The authors should meet all the above conditions.

(B) When a large, multicentre group has conducted the work, the group should identify the individuals who accept direct responsibility for the manuscript. These individuals should fully meet the criteria for authorship defined above.

(C) Acquisition of funding, collection of data, or general supervision of the research group, alone, does not justify authorship.

(D) All persons designated as authors should qualify for authorship, and all those who qualify should be listed.

(E) Each author should have participated sufficiently in the work to take public responsibility for appropriate portions of the content.

(F) All publications (e.g., manuscripts, abstracts, oral/slide presentations, book chapters) based on this study must be submitted to the managing committee for review. The Clinical Study Agreement between the institution, principal investigator, and central trial site will detail the procedures for, and timing of, the committee's review of publications.

Appendix 1: Baseline Questionnaire

Participant Number	

Reduction of serum PFAS levels in Metropolitan Fire Brigade staff via blood and plasma donation

Questionnaire for Participants

Please complete the following questionnaire providing as much detail as possible. Your answers will be kept strictly confidential.

Only the researchers will have access to these details for the purposes of contacting you regarding next steps in the project. Your personal details will be not be linked to your questionnaire responses and will be stored securely as approved by Macquarie University Human Research Ethics Committee. Your individual responses to this questionnaire will never be provided to your employer. MFB will receive reports based on the combined information of all participants in the study.

First Name: ………………………………………………………………………………

Middle Name: ………………………………………………………………………….

Last Name: ………

Residential Address: Number: ………………… Street: ……………………………………………………………

Suburb: ……………………………………………………………… State: ………….. Post Code: …………………

Email Address: ………..

Mobile Telephone Number: ………………………………………………………………..

Name of Next of Kin/Contact Person: ……………………………………………………………………………

Contact Number of Next of Kin/Contact Person: ………………………………………………………………..

Name of General Practitioner: ……………………………………………………………………….

Name of GP Practice: ……………………………………………………………………………………….

Phone number of GP Practice: ……………………………………………………………………………

SECTION A: DEMOGRAPHIC INFORMATION

1. What is your date of birth? ……………………………………..

2. Gender: ☐ Male ☐ Female ☐ Other

3. What is your weight? ……………………………………………..

4. What is your height?...

5. What is your country of birth? …………………………………

6. If not born in Australia what year did you come to live in Australia? ....

SECTION B: LIFESTYLE INFORMATION

7. What is your main source of drinking water?

   ☐ tap water

   ☐ bottled water

   ☐ rain water

   ☐ bore water

SECTION C: BLOOD DONATION

8. Have you ever donated blood? Yes ☐ No ☐

9. (If yes) Are you a current **blood donor**? Yes ☐ No ☐

10. (If yes) How often do you currently donate blood?

    ☐ Less than once a year

    ☐ Once a year

    ☐ 2 to 4 times per year

    ☐ >4 times per year

11. (If yes) When was the approximate **date** of your last blood donation? ……………..

SECTION D: HEALTH INFORMATION

12. Do you currently have any **health problems**?

Please **tick** Yes or No for each condition. If **Yes**, please provide the **details** requested.

**Category**	**Y**	**N**	**Describe**<br>**(date of diagnosis, type of problem and severity)**
Anemia or any blood disorder			
High blood pressure			
Heart disease			
Chronic kidney disease (CKD) or other kidney problems (including kidney stones)			
Thyroid problems			
Liver problems			
Diabetes			
Asthma			
Reproductive or fertility problems			
Serious Arthritis (e.g. Rheumatoid arthritis)			
Cancer			
Other health conditions?			Please list:
Tattoos, piercing in the last 12 months			

13. Are you currently taking any medications? If yes, please list in the table below

Name	Dose	Frequency

14. (If female) Do you have a regular menstrual period (i.e., more than four periods per year)? Yes ☐ No ☐

SECTION E: OCCUPATIONAL HISTORY

15. Please enter the start and stop times in all roles undertaken

**Job title**	**Start month & year**	**Stop month & year (or cont.)**
a. Officer		
b. Firefighter		
c. Instructor (Years as an Instructor)		
d. Mechanical Engineering Workshop Staff		
e. FES staff		
f. Corporate Staff		
g. Other (please specify)		

16. How long have you worked in the firefighting industry (In years, months) ……………………

17. How many years of exposure did you have to aqueous film forming foams (including

    Ansul AFFF and 3M AFFF)? …………………

Have you ever had any **<u>other</u> jobs** (**<u>NOT</u>** at your current employer) in which you were in **contact with PFCs (perfluorinated compounds)** or similar chemicals? (e.g. Firefighter (voluntary, military), facility producing/processing PFCs or similar chemicals, carpet cleaning, retreating carpets or rugs, or professional carpet installer)

Yes ☐ No ☐

If Yes, please provide the **details** requested below:

Job Title	Years in role

<u>Any additional information/medications/medical history etc:</u>

<u>**THANK YOU** FOR TAKING THE TIME TO COMPLETE THIS QUESTIONNAIRE</u>

Acknowledgement: Questionnaire is a modified version of the tool used by Rotander at al. (2015).

Appendix 2: Schedule of assessments

	Screening (pre-intervention)		Baseline									End of intervention period	Follow up
**Assessment**	**Screening -28 days**	**Randomisation 1-3 months**	**Day 0, week 0**	**6 weeks**	**12 weeks**	**18 weeks**	**24 weeks**	**30 weeks**	**36 weeks**	**42 weeks**	**48 weeks**	**52 weeks**	**64 weeks**
Informed consent	X												
Demographics, medical history, concomitant medication (from questionnaire and doctor)	X												
Biochemistry	X											X	
FBC	X											X	
EUC	X											X	
LFT	X											X	
TFTs	X											X	
Lipid profile	X											X	
PFAS blood sample	X		X									X	X
Biobank sample												X	
Blood donation			X		X		X		X		X		
Plasma donation			X	X	X	X	X	X	X	X	X		

All procedures and interventions should be performed at the specified times +/- two weeks.

Appendix 3: Blood samples collection, storage and transport

Blood/Serum for biochemistry testing

Blood/Serum for biochemistry testing- 1 x 8ml SST , 1x4mL EDTA.

Biochemistry test schedule summary:

- Screening
- Post Intervention- 52 weeks

Biochemistry tests include the following:
- Full blood count (FBC)
- Electrolytes, urea, creatine (EUC)
- Liver function tests (LFT)
- Thyroid function tests (TFT)
- Lipid profile.

Blood/Serum for PFAS testing-

Blood/Serum for PFAS testing- 1 x 8ml SST.

***PFAS test schedule summary*:**
- Screening
- baseline - 0 weeks
- post-intervention - 52 weeks
- follow up – 64 weeks.

Samples will be kept cold (4˚ C) and packed securely in eskies during transportation to NATA accredited laboratories for blood biochemistry and PFAS concentrations.

Blood/serum for Biobanking samples (1X 8-10mL SST and 1x10ml EDTA) **will be collected at week 52 visit.**
Biobanking samples transport and processing will be in accordance with the specific Biobank requirements used at that time.

Appendix 4 – PFAS Chemicals Tested

Details of the PFAS chemicals to be tested for are included below.

Analytes and Reporting Limits

Analysis	Acronym	Practical Quantitation Limit (ng/mL)
Perfluorobutanesulfonic acid	PFBS	1
Perfluoropentanesulfonic acid	PFPeS	1
Perfluorohexanesulfonic acid	*PFHxS*	*1*
Perfluoroheptanesulfonic acid	PFHpS	1
Perfluorooctanesulfonic acid	*PFOS*	*1*
Perfluorodecanesulfonic acid	PFDS	2
Perfluorobutanoic acid	PFBA	2
Perfluoropentanoic acid	PFPeA	2
Perfluorohexanoic acid	PFHxA	1
Perfluoroheptanoic acid	PFHpA	1
Perfluorooctanoic acid	*PFOA*	*1*
Perfluorononanoic acid	PFNA	1
Perfluorodecanoic acid	PFDA	2
Perfluoroundecanoic acid	PFUnDA	5
Perfluorododecanoic acid	PFDoDA	5
Perfluorotridecanoic acid	PFTrDA	5
Perfluorotetradecanoic acid	PFTeDA	50
4:2 Fluorotelomer sulfonic acid	4:2 FTS	1
6:2 Fluorotelomer sulfonic acid	6:2 FTS	1
8:2 Fluorotelomer sulfonic acid	8:2 FTS	1
10:2 Fluorotelomer sulfonic acid	10:2 FTS	1
Perfluorooctane sulfonamide	FOSA	10
N-Methyl perfluorooctane sulfonamide	MeFOSA	10
N-Ethyl perfluorooctane sulfonamide	EtFOSA	10
N-Methyl perfluorooctane sulfonamidoethanol	MeFOSE	10
N-Ethyl perfluorooctane sulfonamidoethanol	EtFOSE	50
N-Methyl perfluorooctane sulfonamidoacetic acid	MeFOSAA	2
N-Ethyl perfluorooctane sulfonamidoacetic acid	EtFOSAA	2

Appendix 5 - Advisory panel

Role of the advisory panel: To ensure the safety of the participants in the study.
To provide overall medical, statistical and data integrity oversight of the clinical study.

Members:

1. Dr Robin Gasiorowski.
2. Prof Mark Taylor.
3. A/Prof Merrole Cole-Sinclair.
4. Prof Bruce Lanphear, MD, MPH - Professor, Faculty of Health Sciences, Simon Fraser University, Canada.
5. Dr Roger A. Klein, MA, PhD, MB, BChir, CScie, CChem, FRSC, MIFireE, Cambridge, United Kingdom.
6. Nigel Holmes - Queensland Department of Environment and Heritage Protection, Queensland, Australia.
7. Gabriel Silver.

Frequency of meetings - Meet regularly (every 3 months) to review adverse events related to the clinical study. Minutes from the meetings with highlighted action items will be reported to the investigators team.

Appendix 6 – Briefing Document for GP's for distribution of PFAS test results

The briefing information will use the US National Center for Environmental Health, Agency for Toxic Substances and Disease Registry document: "An Overview of Perfluoroalkyl and Polyfluoroalkyl Substances and Interim Guidance for Clinicians Responding to Patient Exposure Concerns" (ATSDR 2017).

References

Agency for Toxic Substances and Disease Registry (ATSDR) (2017). An Overview of Perfluoroalkyl and Polyfluoroalkyl Substances and Interim Guidance for Clinicians Responding to Patient Exposure Concerns. Available at: https://www.atsdr.cdc.gov/pfc/docs/pfas_clinician_fact_sheet_508.pdf (accessed 14 November 2018).

Agency for Toxic Substances and Disease Registry (ATSDR) (2018). Toxicological Profile for Perfluoroalkyls. Draft for Public Comment (June 2018). US Department of Health and Human Services, Agency for Toxic Substances and Disease Registry (ASTDR) (#CS274127-A). Available at: https://www.atsdr.cdc.gov/toxprofiles/tp.asp?id=1117&tid=237 (accessed 7 November 2018).

Chang, E,T,, Adami, H-O., Boffetta, P,, Cole, P., Starr, T.B., Mandel, J.S. (2014). A critical review of perfluorooctanoate and perfluorooctanesulfonate exposure and cancer risk in humans. *Critical Reviews in Toxicology*, 44(Suppl 1),1-81.

Genuis, S.J., Liu, Y., Genuis, Q.I.T., Martin, J.W. (2014). Phlebotomy Treatment for Elimination of Perfluoroalkyl Acids in a Highly Exposed Family: A Retrospective Case-Series. *PLOS ONE*, 9(12), e114295. Available at: https://doi.org/10.1371/journal.pone.0114295 (accessed 7 November 2018).

Gleason. J.A., Post, G.B., Fagliano, J.A. (2015). Associations of perfluorinated chemical serum concentrations and biomarkers of liver function and uric acid in the US population (NHANES), 2007–2010. *Environmental Research*, 136, 8-14.

International Agency for Research on Cancer (IARC) (2018). Perfluorooctanic acid. International Agency for Research on Cancer Monograph. Available at: https://monographs.iarc.fr/wp-content/uploads/2018/06/mono110-01.pdf (accessed 7 November 2018).

Jones, P.D., Hu, W., De Coen, W., Newsted, J.L., Giesy, J.P. (2009). Binding of perfluorinated fatty acids to serum proteins. *Environmental Toxicology and Chemistry*, 22(11), 2639-49.

Kato, K. Lee-Yang, W., Jia, L.T., Kuklenyik, Z., Calafat, A.M. (2011). Trends in Exposure to Polyfluoroalkyl Chemicals in the U.S. Population: 1999-2008. *Environmental Science & Technology*, 45, 8037-8045.

Kim, S.-J., Heo, S.-H., Lee, D.-S., Hwang, I.G., Lee Y.-B., Cho, H.-Y. (2016). Gender differences in pharmacokinetics and tissue distribution of 3 perfluoroalkyl and polyfluoroalkyl substances in rats. *Food and Chemical Toxicology*, 97, 243-55.

Kirk, M., Smurthwaite, K., Bräunig, J., Trevenar, S., D'Este, C., Lucas, R., Lal, A, Korda, R., Clements, A., Mueller, J., Armstrong, B. (2018). The PFAS Health Study: Systematic Literature Review. Canberra: The Australian National University. Available at: https://rsph.anu.edu.au/files/PFAS%20Health%20Study%20Systematic%20Review.pdf https://www.atsdr.cdc.gov/toxprofiles/tp.asp?id=1117&tid=237 (accessed 7 November 2018).

Knox, S.S., Jackson, T., Frisbee, S.J., Javins, B., Ducatman, A.M. (2011). Perfluorocarbon exposure, gender and thyroid function in the C8 Health Project. *The Journal of Toxicological Sciences*, 36(4), 403-410.

Lorber, M., Eaglesham, G.E., Hobson, P., Toms, L.M.L., Mueller, J.F., Thompson, J.S. 2015. The effect of ongoing blood loss on human serum concentrations of perfluorinated acids. *Chemosphere*, 118:170-177.

Olsen, G.W., Burris, J.M., Ehresman, D.J., Froehlich, J.W., Seacat, A.M., Butenhoff, J.L., Zobel, L.R. (2007) Half-life of serum elimination of Perfluorooctanesulfonate, Perfluorohexanesulfonate, and Perfluorooctanoate in retired fluorochemical production workers. *Environmental Health Perspectives*. 115(9), 1298-305.

Steenland, K., Tinker, S., Frisbee, S., Ducatman, A., Vaccarino, V. (2009). Association of Perfluorooctanoic Acid and Perfluorooctane Sulfonate With Serum Lipids Among Adults Living Near a Chemical Plant. *American Journal of Epidemiology*, 170(10), 1268-1278.

Rappazzo, K. M., Coffman, E., & Hines, E. P. (2017). Exposure to Perfluorinated Alkyl Substances and Health Outcomes in Children: A Systematic Review of the Epidemiologic Literature. *International Journal of Environmental Research and Public Health*, *14*(7), 691. doi:10.3390/ijerph14070691.

Rotander, A., Toms, L-M.L., Aylward, L., Kay, M., Mueller, J.F. (2015). Elevated levels of PFOS and PFHxS in MFB staffs exposed to aqueous film forming foam (AFFF). *Environment International*, 82, 28-34.

Toms, L.-M.L., Thompson, J., Rotander, A., Hobson, P., Calafat, A.M., Kato, K., Ye, X., Broomhall, S., Harden, F., Mueller, J.F. (2014). Decline in perfluorooctane sulfonate and perfluorooctanoate serum concentrations in an Australian population from 2002 to 2011. *Environment International*, 71, 74-80.

United States Environmental Protection Agency (USEPA) (2018). Research on Per- and Polyfluoroalkyl Substances (PFAS). United States Environmental Protection Agency. Available at https://www.epa.gov/chemical-research/research-and-polyfluoroalkyl-substances-pfas (accessed 9 November 2018).

Wong, F., MacLeod, M., Mueller, J.F., Cousins, I.T. (2014). Enhanced Elimination of Perfluorooctane Sulfonic Acid by Menstruating Women: Evidence from Population-Based Pharmacokinetic Modeling. *Environmental Science & Technology*, 48(15), 8807-8814.

Zhang, Y., Beesoon, S., Zhu, L., Martin, J.W. (2013). Biomonitoring of perfluoroalkyl acids in human urine and estimates of biological half-life. *Environmental Science & Technology*, 47(18), 10619-10627.
