# Supplementary material for: Effect of Plasma and Blood Donations on Levels of Perfluoroalkyl and Polyfluoroalkyl Substances in Firefighters in Australia: A Randomized Clinical Trial
Source: JAMA Netw Open. 2022 Apr 8;5(4):e226257. doi: 10.1001/jamanetworkopen.2022.6257 (PMC8994130; doi:10.1001/jamanetworkopen.2022.6257)
Supplement: Supplement 2. — eMethods. eResults. eFigure 1. Association Between Screening and Week 52 Levels of TSH eFigure 2. Association Between Screening and Week 52 Levels of ALT eFigure 3. Association Between Screening and Week 52 Levels of Platelets eTable. Adverse Events eFigure 4. Per-Protocol Analysis eAppendix. [file jamanetwopen-e226257-s002.pdf]

Supplementary Online Content

Gasiorowski R, Forbes MK, Silver G, et al. Effect of plasma and blood donations on levels of perfluoroalkyl and polyfluoroalkyl substances in firefighters in Australia: a randomized clinical trial. *JAMA Netw Open.* 2022;5(4):e226257. doi:10.1001/jamanetworkopen.2022.6257

eMethods.
eResults.
eFigure 1. Association Between Screening and Week 52 Levels of TSH
eFigure 2. Association Between Screening and Week 52 Levels of ALT
eFigure 3. Association Between Screening and Week 52 Levels of Platelets
eTable. Adverse Events
eFigure 4. Per-Protocol Analysis
eAppendix.

This supplementary material has been provided by the authors to give readers additional information about their work.

© 2022 Gasiorowski R et al. *JAMA Network Open.*

eMethods.

Further detail on statistical analyses

All primary and secondary endpoints were continuous variables and substantial outliers were winsorized to 3 standard deviations from the mean. Remaining minor to moderate non-normality was handled in the reported analyses using bootstrapping (5000 random bootstraps resampling with replacement) to derive robust estimates of standard errors, confidence intervals and *p*-values. Interactions between screening or baseline levels of each endpoint with the treatment group covariate were examined using the Johnson-Neyman technique.[19] Where interaction effects were small ($\Delta R^2 < 0{\cdot}05$) and the Johnson-Neyman bounds were outside of the observed range of values, the interaction effects were not included in the final model and interpretation of the differences between treatment groups were restricted to the observed range in our sample at baseline.

Sensitivity analyses were conducted for the primary endpoints using natural log transformations to handle non-normality and using multiple imputation (with 20 imputations) to handle the small amount of missing data (maximum of n = 18 [6·3%] missing data at week 64). Sensitivity analyses were also conducted recoding all PFAS levels below instrument detection limits at the upper bound of the threshold (e.g., PFHxS values < 1 ng/mL were treated as 1 ng/mL instead of 0 ng/mL). All sensitivity analyses used the winsorized data and were nearly identical in interpretation, both in terms of effect sizes and significance levels.

Sensitivity analysis

The only difference in interpretation between the primary analyses and all sensitivity analyses conducted was that the increase in PFHxS in the observation group from baseline to week 52 was statistically significant for both the multiple imputation and log transformation sensitivity analyses, p = 0·036 and p < 0·0001 respectively. However, this slight increase is less than changes in concentrations (i.e. < 1·0 ng/mL) that can be measured reliably in an individual.

© 2022 Gasiorowski R et al. *JAMA Network Open.*

eResults.

Secondary endpoints

The following endpoints had non-significant differences between treatment groups at week 52, controlling for screening levels, associated with conventionally "trivial" standardised effect sizes ($p > 0{\cdot}05$, $\eta_p^2 < 0{\cdot}01$): Total cholesterol, LDL, T4, T3, GGT, albumin, sodium, potassium, chloride, urea, ALP, and lymphocytes.

The following endpoints had statistically non-significant differences between treatment groups associated with conventionally "small" standardised effect sizes ($p > 0{\cdot}05$, $0{\cdot}1 \leq \eta_p^2 < 0{\cdot}2$): HDL, triglycerides, bilirubin, creatinine, white blood cell count, and neutrophils.

TSH, ALT, and platelets had no significant main effects for randomisation group, ($p > 0{\cdot}05$ and $0{\cdot}01 < \eta_p^2 < 0$ .02) but had significant group*screening level interactions. The Johnson-Neyman technique subsequently indicated group-level differences at low and high levels of all three endpoints.

For individuals with higher levels of TSH at screening (TSH > 1·1 mIU/L), there were group-level differences in mean TSH levels at week 52 (eFigure 1). Plasma donation was associated with larger mean increases in TSH than blood donation or observation; the opposite pattern was found for individuals with very low levels of TSH at screening (TSH < 0·6 mIU/L).

For individuals with higher levels of alanine aminotransferase (ALT) at screening (ALT > 37 U/L), there were group-level differences in mean ALT levels at week 52 (eFigure 2). Plasma donation was associated with larger mean decreases in ALT than blood donation or observation; the opposite pattern was found for individuals with very low levels of ALT at screening (ALT < 15 U/L).

For individuals with higher levels of platelets at screening (platelets > 323 $x10^9$/L), there were group-level differences in mean platelet levels at week 52 (eFigure 3). Blood and plasma donation were associated with larger mean decreases in platelets than observation; the opposite pattern was found for individuals with lower levels of platelets at screening (platelets < 227 x 10^9/L).

© 2022 Gasiorowski R et al. *JAMA Network Open.*

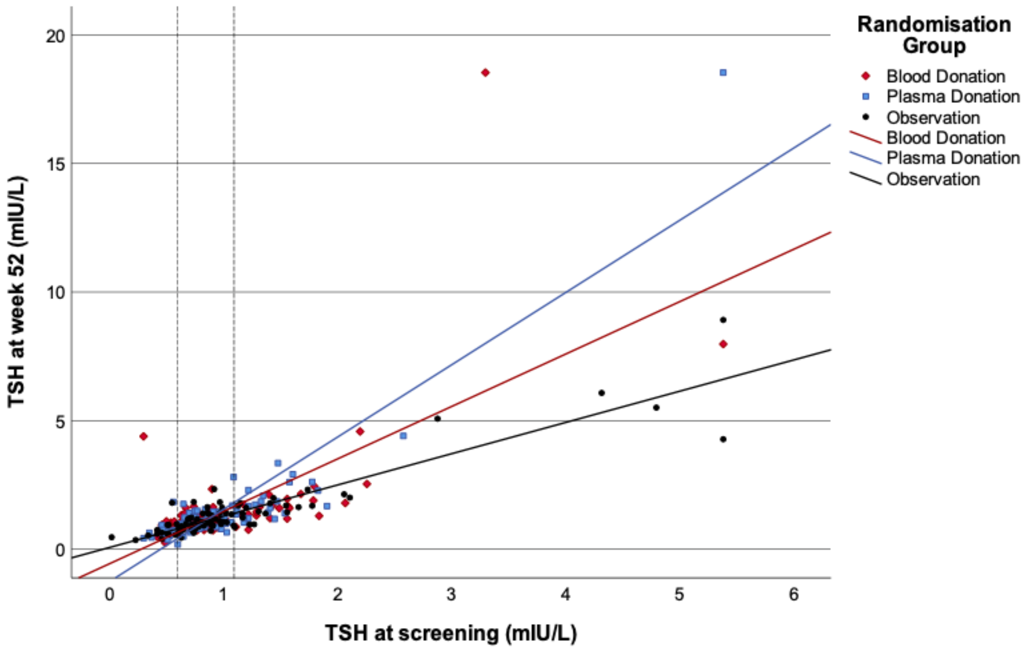

eFigure 1. Association Between Screening and Week 52 Levels of TSH

Johnson-Neyman boundaries of significance lie outside of the dashed lines where shallower slopes indicate stronger treatment effects.

© 2022 Gasiorowski R et al. *JAMA Network Open.*

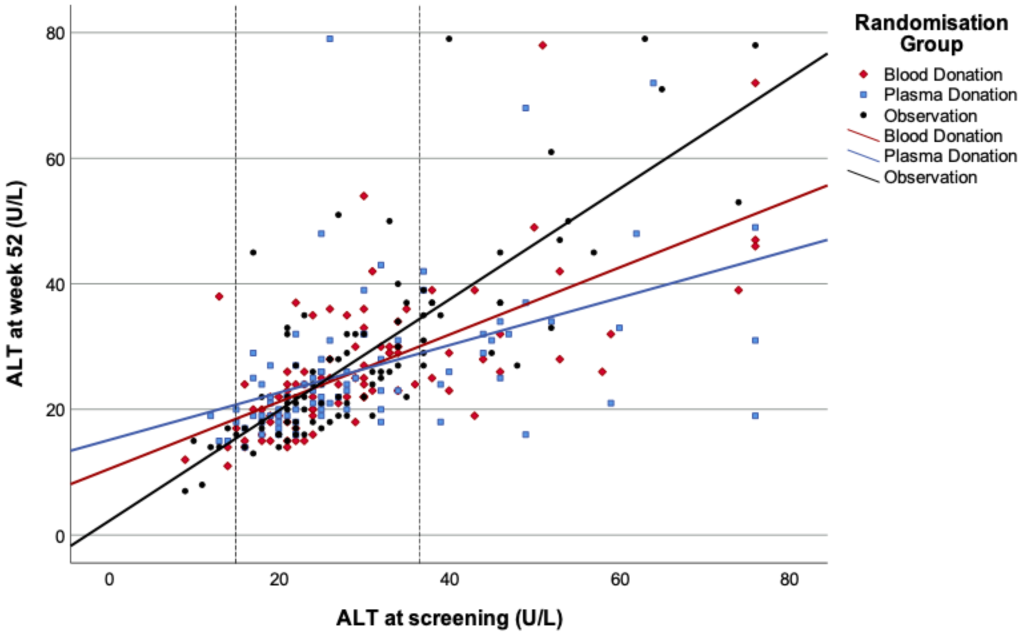

eFigure 2. Association Between Screening and Week 52 Levels of ALT

Johnson-Neyman boundaries of significance lie outside of the dashed lines where shallower slopes indicate stronger treatment effects.

© 2022 Gasiorowski R et al. *JAMA Network Open.*

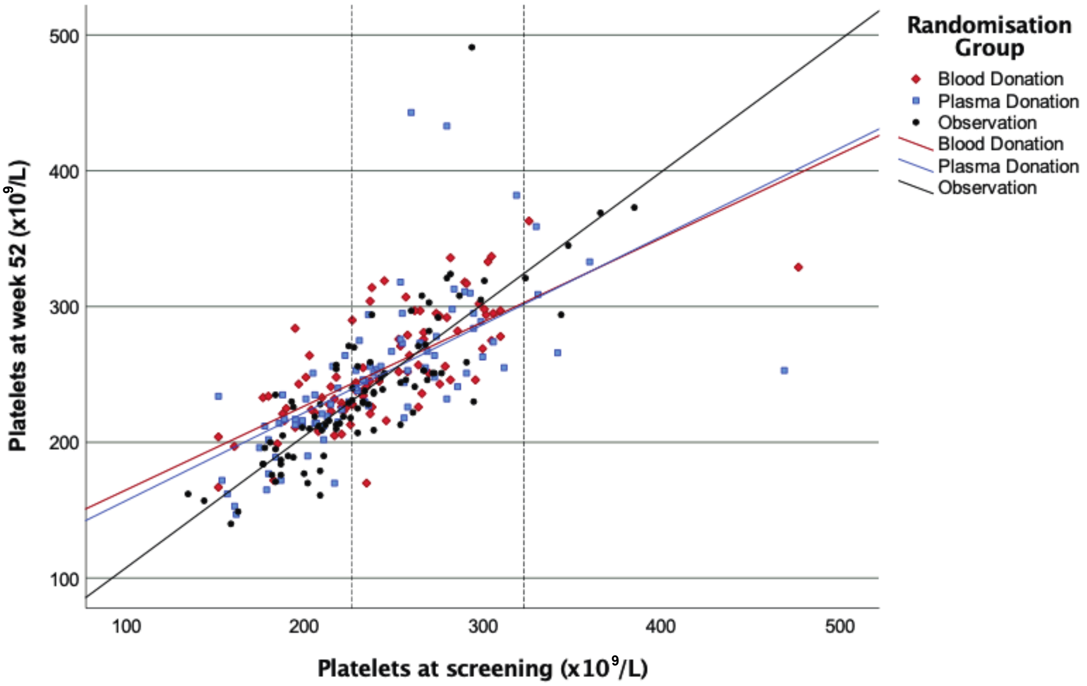

eFigure 3. Association Between Screening and Week 52 Levels of Platelets

Johnson-Neyman boundaries of significance lie outside of the dashed lines where shallower slopes indicate stronger treatment effects.

© 2022 Gasiorowski R et al. *JAMA Network Open.*

eTable. Adverse Events

n (% of participants within group)

	Plasma group (n = 95)				Blood donation group (n = 95)			
	**Grade 1**	**Grade 2**	**Grade 3**	**Grade 4-5**	**Grade 1**	**Grade 2**	**Grade 3**	**Grade 4-5**
Any	7 (7·4%)	0	1 (1·1%)	0	4 (4·2%)	0	0	0
Haematoma	5 (5·3%)	0	0	0	2 (2·1%)	0	0	0
Low ferritin	0	0			2 (2·1%)	0	0	0
Plasma donation reaction	0	0	1 (1·1%)	0	0	0	0	0
Pain	1 (1·1%)	0	0	0	0	0	0	0
Nausea	1 (1·1%)	0	0	0	0	0	0	0

© 2022 Gasiorowski R et al. *JAMA Network Open.*

eFigure 4. Per-Protocol Analysis

Intention to treat (solid lines) versus per protocol (dashed lines) analyses of observed mean changes in A) PFOS and B) PFHxS levels from baseline to week 52. Error bars represent the standard error of the mean

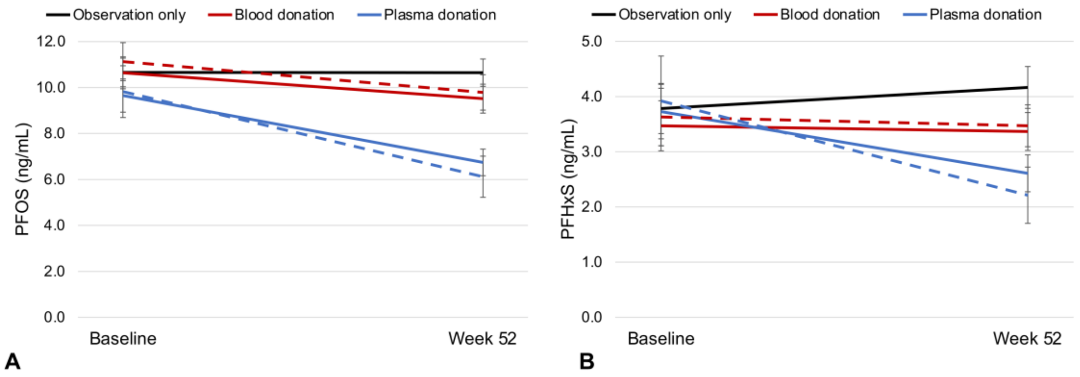

© 2022 Gasiorowski R et al. *JAMA Network Open.*

eAppendix.

Appendix 1 PFAS Analysis

Blood PFAS were analysed by Envirolab Services Pty Ltd by combining liquid chromatography with mass spectrometry (LC-MS/MS). Envirolab are accredited by the National Association of Testing Authorities (NATA) to perform this testing. The following PFAS were measured.

Perfluorooctanesulfonic acid - PFOS
Perfluorohexanesulfonic acid - PFHxS
Perfluorooctanoic acid - PFOA
Perfluorotridecanoic acid
Perfluorododecanoic acid
Perfluoroundecanoic acid
Perfluorodecanoic acid
Perfluorononanoic acid
Perfluoroheptanoic acid
Perfluorohexanoic acid
Perfluoropentanoic acid
Perfluorobutanoic acid
Perfluorodecanesulfonic acid
Perfluoroheptanesulfonic acid
Perfluoropentanesulfonic acid
Perfluorobutanesulfonic acid
Perfluorooctane sulfonamide
Perfluorotetradecanoic acid
10:2 FTS
8:2 FTS
6:2 FTS
4:2 FTS
EtPerfluorooctanesulf- amid oacetic acid
MePerfluorooctanesulf- amid oacetic acid
N-Et perfluorooctanesulfonamid oethanol
N-Me perfluorooctanesulfonamid oethanol
N-Ethyl perfluorooctanesulfon amide
N-Methyl perfluorooctane sulfonamide

© 2022 Gasiorowski R et al. *JAMA Network Open.*
