# Supplementary material for: Effect of Plasma and Blood Donations on Levels of Perfluoroalkyl and Polyfluoroalkyl Substances in Firefighters in Australia: A Randomized Clinical Trial
Source: JAMA Netw Open. 2022 Apr 8;5(4):e226257. doi: 10.1001/jamanetworkopen.2022.6257 (PMC8994130; doi:10.1001/jamanetworkopen.2022.6257)
Supplement: Supplement 3. — Data Sharing Statement [file jamanetwopen-e226257-s003.pdf]

Data Sharing Statement

Gasiorowski. Effect of Plasma and Blood Donations on Levels of Perfluoroalkyl and Polyfluoroalkyl Substances in Firefighters in Australia. *JAMA Netw Open*. Published April 08, 2022. doi:10.1001/jamanetworkopen.2022.6257

Data
Data available: Yes
Data types: Deidentified participant data
How to access data: The de-identified participant data is available upon written request sent to the corresponding author. Note: Human Research Ethics Committee/IRB approval and signed data access agreement will be required.
When available: With publication

Supporting Documents
Document types: None

Additional Information
Who can access the data: Researchers whose proposed use of the data has been approved
Types of analyses: Any purpose with Human Research Ethics Committee/IRB approval.
Mechanisms of data availability: With a signed data access agreement
